# Cumin Seed Oil Induces Oxidative Stress-Based Antifungal Activities on *Fusarium graminearum*

**DOI:** 10.3390/pathogens13050395

**Published:** 2024-05-08

**Authors:** Emre Yörük, Zeynep Danışman, Murat Pekmez, Tapani Yli-Mattila

**Affiliations:** 1 Department of Molecular Biology and Genetics, Faculty of Arts and Sciences, Istanbul Yeni Yuzyil University, Cevizlibag, Istanbul 34010, Turkey; emre.yoruk@yeniyuzyil.edu.tr; 2 Programme of Molecular Biotechnology and Genetics, Institute of Graduate Studies in Sciences, Istanbul University, Suleymaniye, Istanbul 34116, Turkey; zeynepdanismanx@gmail.com; 3 Department of Molecular Biology and Genetics, Faculty of Sciences, Istanbul University, Vezneciler, Istanbul 34134, Turkey; mpekmez@istanbul.edu.tr; 4 Department of Life Technologies/Molecular Plant Biology, University of Turku, FI-20520 Turku, Finland

**Keywords:** cumin seed oil, *Fusarium* head blight, gene expression, oxidative stress, polymorphisms

## Abstract

In this study, the antifungal activity of cumin seed oil (CSO) was tested on *Fusarium graminearum*. (i) Minimum inhibitory concentrations (MICs) and related concentrations (IC_75_, IC_50_, and IC_25_) were detected; (ii) toxicity was evaluated by a water-soluble tetrazolium salt-1 (WST-1) assay; (iii) genomic/epigenomic alterations were evaluated by the coupled restriction enzyme digestion-random amplification (CRED-RA) method; (iv) oxidative stress was investigated by *CAT* expression, catalase activity, and DCF-DA staining; (v) deoxynivalenol biosynthesis was evaluated by *tri6* expression; (vi) and potential effects of CSO on wheat were tested by a water loss rate (WLR) assay. MIC, IC_75_, IC_50_ and IC_25_ values were detected at 0.5, 0.375, 0.25, and 0.125 mg mL^−1^. In WST-1 assays, significant decreases (*p* < 0.001) were detected. Genomic template stability (GTS) related to methylation differences ranged from 94.60% to 96.30%. Percentage polymorphism for *Hap*II/*Msp*I values were as 9.1%/15.8%. *CAT* (oxidative stress-related catalase) and *tri6* (zinc finger motif transcription factor) gene expressions were recorded between 5.29 ± 0.74 and 0.46 ± 0.10 (*p* < 0.05). Increased catalase activity was detected (*p* < 0.05) by spectrophotometric assays. DCF-DA-stained (oxidative stressed) cells were increased in response to increased concentrations, and there were no significant changes in WLR values. It was concluded that CSO showed strong antifungal activity on *F. graminearum* via different physiological levels.

## 1. Introduction

*Fusarium* head blight (FHB) is one of the most destructive and devastating fungal diseases of small grain cereals worldwide. Epidemics result in reduction in crop quantity and quality. Consequently, economic losses reaching up to billions of dollars have been reported in many regions throughout the world [1,2,3]. In addition to yield losses, mycotoxin contamination also occurs in wheat and barley fields where FHB present. Deoxynivalenol, nivalenol, and their acetylated derivatives have been reported as the predominating mycotoxins accumulated on small grain cereals infected with FHB causal agents, predominantly *Fusarium graminearum* [4,5,6,7].

Causal agents of FHB show variation due to several factors such as population dynamics of phytopathogens, climatic conditions, and crop rotations. However, *F. graminearum* sensu lato seems to be a predominating causal agent of FHB in particularly humid and semi-humid regions [3,8,9]. *F. graminearum*, with more than 10 members, has been accepted as a species complex nowadays and the global member of this species complex is *F. graminearum* sensu stricto [9,10,11]. *F. graminearum* is a homothallic phytopathogen and it primarily infects wheat, maize, and barley worldwide. Also, this species complex produces several important mycotoxins such as deoxynivaleol, nivalenol, and zearalenone [4,12]. *F. graminearum* is included in the list of most important phytopathogens worldwide [13]. This fungus could also be considered as a model organism, and there are more than 100 genome assembly and annotation reports belonging to different strains deposited under GenBank, and at least three main genetic populations of *F. graminearum* in the northern hemisphere [14].

Development of disease resistant plant cultivars, usage of antagonistic microorganisms, and fungicide treatment have been widely used for in vitro and in planta studies to manage FHB. Each strategy has several disadvantages. Among these strategies, common fungicide treatment such as carbendazim and tetraconazole result in effective management against FHB and *F. graminearum* in many regions. However, it seems that fungicide resistance will become one of the most important topics in plant disease research in the near future [15,16,17,18,19,20,21,22,23,24]. Especially, resistance to *β-tubulin*- and *cyp51*-targeting fungicides in *Fusarium* spp. seem to be even more important for agricultural sustainability for the near future. On the other hand, researchers have focused on working with alternative specific metabolites, which could be used in fighting against *F. graminearum* [25,26,27,28,29,30]. These studies have been conducted using mainly phenotypic and transcript analysis. Further investigations could especially include epigenetics analysis, as well as fungicide and plant-derived essential oil mixtures for *F. graminearum* and related phytopathogens.

Since antimicrobial resistance remains an important problem for agriculture, researchers try to discover or develop novel antimicrobial compounds. Cumin (*Cuminum cyminum* L.) is well known for its beneficial effects, such as antibacterial, anti-cancerous and anti-diabetic effects, and natural essential oil extracts of CSO include useful and pharmaceutically important compounds, including cuminal and cuminic alcohol [31,32]. However, detailed knowledge related to the mechanisms behind these useful effects of CSO is missing from the literature. In this study, the potential antifungal activity of cumin (*Cuminum cyminum* L.) seed oil (CSO) on *F. graminearum* was investigated. It was mainly aimed at revealing the (i) potential antifungal effects of CSO on *F. graminearum* and (ii) the mechanisms underlying the potential antifungal effects of CSO versus *F. graminearum*. In the current study, the effect of CSO on the reference strain PH-1 of *F. graminearum* was investigated in terms of phenotypic, genomic, epigenomic, transcript, and antioxidant levels for the first time.

## 2. Materials and Methods

### 2.1. Antimicrobial Assay and Cell Proliferation Test

A *Fusarium graminearum* PH-1 reference strain was used in this study. The fungal strain was grown on a potato dextrose agar (PDA) at 26 ± 2 °C for 7 days. CSO, including eugenol (0.5%) and limonene (0.4%) (W234300-Sigma, Saint Louis, MO, USA), was dissolved in ethanol. For minimum inhibition concentration (MIC) and related inhibition concentration determination, preliminary studies included different concentrations of CSO with logarithmic series of 2, 5, and 10 µg mL^−1^. Mycelium discs (0.25 cm^2^) from 7-day-old fresh fungal cultures were transferred onto a PDA medium with different concentrations of CSO (0, 0.125, 0.25, 0.375, and 0.5 mg mL^−1^) from CSO stocks dissolved in ethanol, with a concentration of 1mg mL^−1^. The effect of the CSO was analyzed according to the linear growth rate measurements obtained on the 4th and 7th day of incubation. The growth inhibition as a percent (minimum inhibition concentration [MIC], growth inhibition at 75% [IC_75_], half inhibition [IC_50_], and growth inhibiton at 25% [IC_25_]) was calculated in comparison to the control set.

The evaluation of cell proliferation was determined by a WST-1 (Roche, Basel, Switzerland) analysis. One cm^2^ discs of control and experimental groups were dissolved in phosphate-buffered saline (PBS). A total of 10 μL WST-1 was added onto 100 μL cells suspended in PBS, and samples were incubated for 3 h at 28 °C in the dark. Spectrophotometric measurements were recorded at 450 and 620 nm wavelengths after incubation. The background radiation was excluded via the ∆_450–620_ nm calculation. The cytotoxicity level due to CSO treatment was evaluated by a comparison of absorbance values from the control and experiment sets.

### 2.2. Nucleic Acid Isolation and cDNA Synthesis

The genomic DNA (gDNA) from the control and experiment sets was extracted using a commercial genomic DNA isolation kit (Anatolia Geneworks, Istanbul, Turkey) following the experimental procedures provided by the manufacturer. The quality of gDNA was checked using 0.8% agarose gels. The concentration and purity of gDNA were analyzed by a spectrophotometer (Nanodrop-Thermo, Waltham, MA, USA).

The total RNA isolation from the control and experimental groups of *F. graminearum* PH-1 was performed using the monophasic reagent Hibrizol following the manufacturer’s recommendations (Hibrigen, Istanbul, Turkey). The isolated RNA was analyzed with 0.8% agarose gels and spectrophotometric measurements.

The synthesis of cDNA from RNA molecules was carried out using the first strand cDNA synthesis kit (Takara, Shiga, Kansai, Japan). According to the recommendations from the manufacturer, 50 µM Oligo dT, 50 µM Random 6mer, 1× Buffer, 200 U Reverse Transcriptase enzyme, and total RNA molecules equal to 2 µg RNA were combined in 20 µL total volume. cDNA was synthesized by incubating samples at 37 °C for 20 min and 85 °C for 5 min. cDNAs were diluted by ¼ and then used in qPCR assays.

### 2.3. RAPD (Randomly Amplified Polymorphic DNA) and Coupled Restriction Enzyme Digestion-Random Amplification (CRED-RA) Assays

RAPD/CRED-RA techniques were used for the examination of the methylation status of the genome and template stability in response to the CSO treatment. The final concentrations of the components in the PCR assays were adjusted by 50 ng gDNA, 1× PCR buffer, 3 mM MgCl2, 0.4 mM dNTP mix, 10 pmol 10-mer primer, and 0.04 U μL^−1^ *Taq* DNA polymerase in a volume of 25 µL. In total, 23 common RAPD primers (Eurofins, Saint-Augustin, France) were used in this study (Table 1). Reaction was performed in two stages, following the pre-denaturation process at 94 °C for 2 min. Four loops in the first stage included incubation at 94 °C for 1.5 min, at 37 °C for 1.5 min, and at 72 °C for 3 min. The next 36 loops were performed for 1 min at 94 °C, 1 min at 37 ºC, and 2 min at 72 °C. The final elongation stage was carried out as 10 min at 72 °C. RAPD bands were run on 1.8% agarose gels and visualized under UV light [33].

The genomic template stability (GTS %) was calculated with the following formula: GTS = (1 − [a/n]) × 100, where a is the average number of polymorphic bands detected in each cumin-treated sample, and n is the number of total bands in the control set. Resolving power (RP) values for each primer were calculated using common formulas developed by Prevost and Wilkinson [34].

In CRED-RA assays, the gDNAs of the control and experimental groups were digested using *Hap*II and *Msp*I restriction endonucleases (Takara, Japan). The digestion reactions were combined in a total volume of 50 μL using 500 ng gDNA, 20 U HapII or MspI enzyme, 0.01% BSA, and 1× digestion buffer, and the mixture was incubated for 1 h 30 min at 37 °C. A stop reaction was carried out by adding 1× loading buffer and left at room temperature for 5 min. The digestion products were used for RAPD analysis as described before. The polymorphism value (%) was determined according to the formula: Polymorphism % = (5 − mC/5 + mC + C) × 100, where C is cytosine and mC is methylated cytosine [35].

### 2.4. Gene Expression Analysis

Fold changes in gene expression levels between control and experimental groups as a result of CSO treatment were determined by qPCR analysis. Genes associated with oxidative stress response (CAT: catalase, which has only one splice variant and the biological function was proved to be oxidative stress response [catalase_3: IPR018028/FGSG_06733]) and deoxynivalenol biosynthesis (tri6: a zinc finger transcription factor) were chosen as the target genes. β-tubulin was used as a housekeeping gene. qPCR assays were performed using the Sybr Green I fluorescent dye. qPCRs were conducted in a reaction volume of 20 μL containing 1× Sybr Green I mixture (Episozyme, Istanbul, Turkey), 5 pmol forward/reverse primers developed by Gazdagli et al. [25], and an amount of cDNA corresponding to 50 ng RNA. The cycling conditions, melting curve analysis, and fold change determination analysis were carried out as reported by Gazdagli et al. [25].

### 2.5. Intracellular ROS Detection by Fluorescence Microscopy

Changes related to oxidative stress that could occur in the experimental groups depending on the CSO treatment were revealed by fluorescence microscopy observations. For these purposes, carboxy methyl cellulose (CMC) liquid cultures were obtained. Up to seven 0.25 cm^2^ fungal discs were transferred to a CMC broth medium of 25 mL and grown at 26 ± 2 °C for 7 days in a rotary shaker at 120 rpm. 1 × 10^5^ spore mL^−1^ were used in fluorescence analysis. The presence of potential oxidative stress/reactive oxygen species (ROS) was investigated by 2′,7′-dichlorofluorescin diacetate (DCF-DA) staining. Fungal cultures were incubated for 30 min at 25 °C using 4% formaldehyde and 0.1% Triton-X 100 to fix the samples. Fixed cells were washed twice with 1× PBS. Fluorescent staining was performed to determine the presence and status of ROS (DCF-DA; 5 μg mL^−1^) after washing. DCF-DA fluorescence staining was visualized with FITCH filter [28,29,36]. ROS detection for potential oxidative stress response in *F. graminearum* was carried out via a PH2 condenser with excitation of 494 nm and emission of 518 nm.

### 2.6. Assessment of Catalase (CAT) Activity

Total protein from the control and experiment sets was extracted from 0.5 g of fresh mycelium using a common phosphate buffer protocol [37]. Protein concentration was detected using bovine serum albumin standards series by using a bicinchoninic acid assay (BCA) protein assay kit following the manufacturer’s recommendations (Atlas, Ankara, Turkey). An equalized protein concentration (1 mg) was used to take the kinetic measurement at 240 nm for detecting catalase activity. A reaction mix (112.2 μL phosphate buffer (50 mM NaH2PO4; pH 7.2) and 80 μL H_2_O_2_ (40 mM)) were incubated at 30 °C for 2.5 min. After 6.8 μL protein extract was added to the reaction mix, the kinetic measurement was recorded at 10 s intervals for 2 min. The difference in absorbance through time (Δ_240_) was used for inference of the catalase activity. Fold changes in CAT activity were calculated via normalization by control series.

### 2.7. Water Loss Rate Assay

The potential adverse effect of CSO on wheat was investigated via water loss rate (WLR) analysis. For this purpose, seeds of *Triticum aestivum* L. cv. Ceyhan 99 were used in WLR assays. The protocol provided by Suprunova et al. [38] was followed. A total of 10 seeds for each technical repeat were germinated on moist petri dishes at room temperature for seven days. The 7-day-old shoots with roots were transferred to plastic boxes containing soil. The plantlets were irrigated with distilled water and the CSO with 0.5 mg mL^−1^ for 7 days. The samples were incubated at 25 ± 2 °C. The expanded leaves of the 14-day-old plants were cut and the fresh weight (FW) was measured. The leaves were left on filter paper for 24 h, and then weight (W24) was measured. The leaves were left at 65 °C for 24 h and the dry weight (DW) was recorded. The WLR was calculated as following the formula: WLR (gh^−1^ g^−1^ DW) = (FW − W24)/(DW × 24).

### 2.8. Statistical Analysis

Each experimental procedure included two technical and three biological repeats. For statistical analyses, a *t*-test, one-way and two-way analysis of variance (ANOVA) with Tukey’s post-test, normality tests (accompanied by Shapiro–Wilks test/Pearson’s correlation matrix), and PCA analysis were carried out using GraphPad Prism 9.0 (Dotmatics, La Jolla, CA, USA) and R/R-Studio (Posit-PBC, Boston, MA, USA). The standard deviation for each experiment set was calculated via descriptive/column statistics. The confidence interval was determined at 0.05.

## 3. Results

### 3.1. Antimicrobial Activity and Cell Proliferation Analysis

CSO strongly repressed in vitro fungal growth. The MIC, IC_75_, IC_50_ and IC_25_ values were recorded as 0.5, 0.375, 0.25 and 0.125 mg mL^−1^ CSO treatment on PDA media, respectively. In further analysis, PDA amended with different concentrations of CSO (0, 0.125, 0.25, 0.375, and 0.5 mg mL^−1^), control, IC_25_, IC_50_, and IC_75_, were used.

**Figure 1:**
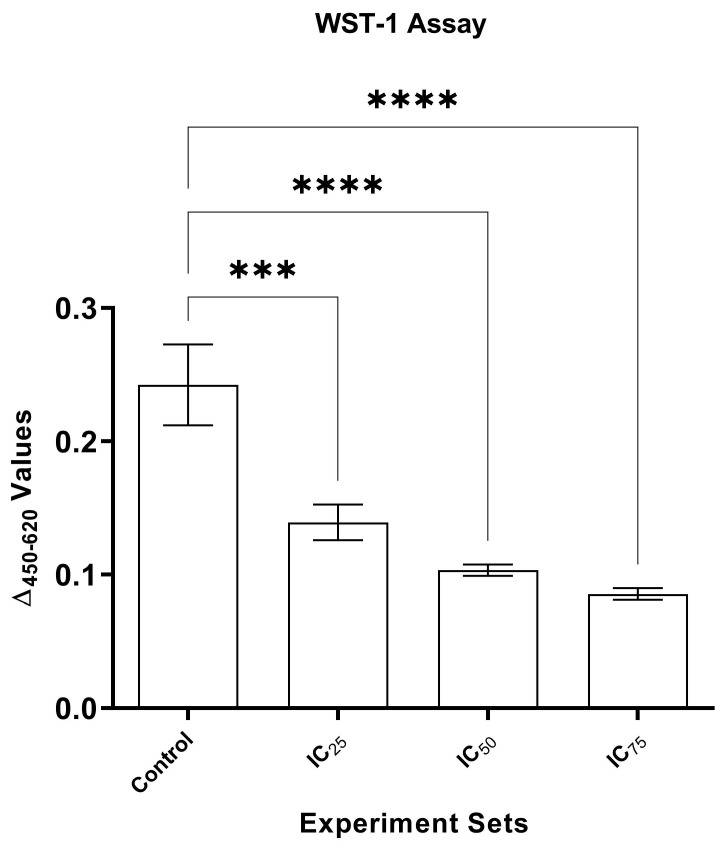
Comparison of WST-1 assay values of experimental sets which were exposed to different concentrations of cumin seed oil. The control, IC_25_, IC_50_, and IC_75_ sets were the strains grown on PDA amended with 0, 0.125, 0.25, and 0.375 µg µL_-1_ cumin seed oil. ***: *p* < 0.001 and ****: *p* < 0.0001.

In the WST-1 assays, significant decreases (*p* < 0.001) were detected in IC_25_, IC_50_, and IC_75_ sets in comparison to the control set via one-way ANOVA analysis. ∆_450–620_ values were recorded as 0.24 ± 0.03, 0.13 ± 0.01, 0.10 ± 0.01, and 0.08 ± 0.01 in the control, IC_25_, IC_50_, and IC_75_ sets, respectively (Figure 1).

### 3.2. RAPD and CRED-RA Analysis

RAPD and CRED-RA analysis were carried out with gDNA molecules obtained from the control, IC_25_, IC_50_, and IC_75_ sets with quality and quantity (Δ_260/280_ = 1.7–1.9 and 0.5–2 µg µL^−1^). All 10-mer RAPD primers gave amplicon(s) from experiment sets. GTS values for IC_25_, IC_50_, and IC_75_ sets were recorded as 94.60%, 95.50% and 96.30%, respectively. RP values were in the range of 1.32 (OPG16) and 5.74 (OPM02) (Table 1). In RAPD analysis, 113 bands were obtained and only 9 of them were polymorphic. In CRED-RA assays, 141 bands were amplified, and 23 bands were idiomorphic. Minimum and maximum band numbers for CRED-RA analysis were recorded as 4 (OPB06) and 11 (OPM02), respectively. The % of polymorphisms for *Hap*II and *Msp*I enzymes ranged between 9.9 and 10.9% and 9.1–15.8%, respectively (Table 2).

### 3.3. Gene Expression Analyses

**Figure 2:**
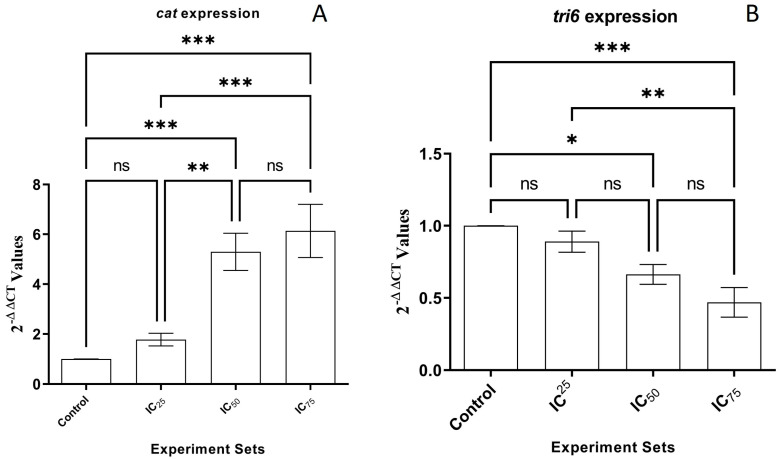
Fold changes in *CAT* (**A**) and *tri6* (**B**) expression. Control, IC_25_, IC_50_ and IC_75_ sets were the strains grown on PDA amended with 0, 0.125, 0.25, and 0.375 µg µL^−1^ cumin seed oil). ns: no significant changes, *: *p* < 0.05, **: *p* < 0.01, and ***: *p* < 0.001.

Fold changes in CAT and tri6 expression were normalized in comparison to the *β-tubulin* expression. Relative transcript abundance for the *CAT* gene in IC_25_, IC_50_, and IC_75_ sets were recorded as 1.77 ± 0.25, 5.59 ± 0.74, and 6.13 ± 1.06, respectively (Figure 2A). There was no statistically significant difference between IC_25_ treatment and the untreated control (*p* > 0.05), whereas the changes in IC_50_ and IC_75_ treatments were statistically significant (*p* ˂ 0.001). Fold changes in the *tri6* expression in IC_25_, IC_50_, and IC_75_ treatments were calculated as 0.89 ± 0.07, 0.66 ± 0.06, and 0.46 ± 0.10, respectively (Figure 2B). Similar to *CAT* expression, there were no significant differences between IC_25_ treatment and the control set in the *tri6* expression (*p* > 0.05). The changes in IC_50_ (*p* ˂ 0.05) and IC_75_ (*p* ˂ 0.001) treatments were as statistically significant by one-way ANOVA analysis.

### 3.4. Fluorescence Microscopy Analysis

**Figure 3:**
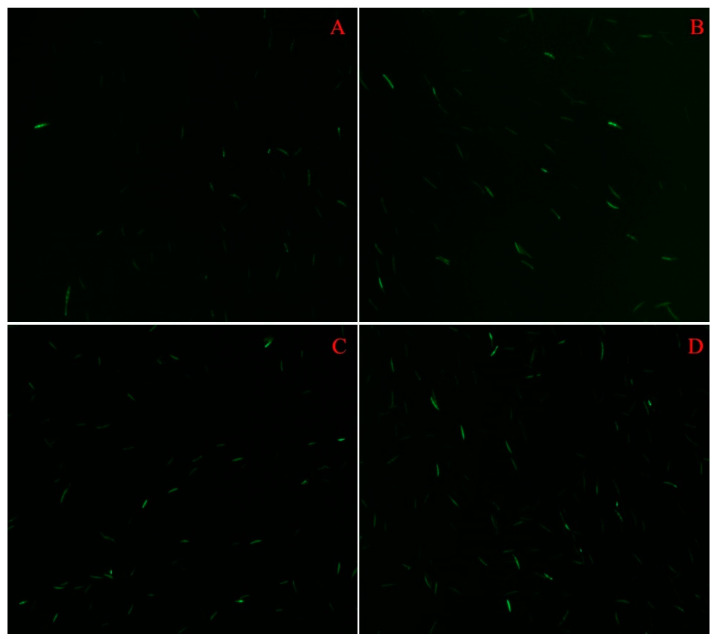
FITCH filter-captured profile of spores treated with different concentrations of cumin seed oil. (**A**): control set, (**B**): IC_25_ set, (**C**): IC_50_ set, and (**D**): IC_75_ set at 20× magnification. Green-coloured cells show the presence of oxidative stress.

The presence of oxidative stress was evaluated via DCF-DA staining in the control, IC_25_, IC_50_, and IC_75_ sets. ROS activity was not detected in most of the spores in the control set, whereas three experimental sets showed ROS activity under a fluorescent microscope. It was seen that the cells of IC_25_, IC_50_, and IC_75_ sets were green colored (Figure 3). The number of spores (100 cell is equalized to “1” value) subjected to oxidative stress for the control, IC_25_, IC_50_, and IC_75_ sets were recorded as 0.08 ± 0.01, 0.20 ± 0.03, 0.49 ± 0.04, and 0.59 ± 0.03, respectively. The changes in ROS activity were significantly different in IC_50_ and IC_75_ sets in comparison to the control set (*p* ˂ 0.001), while no significant alteration was recorded in IC_25_ via one-way ANOVA analysis.

### 3.5. CAT Activity

**Figure 4:**
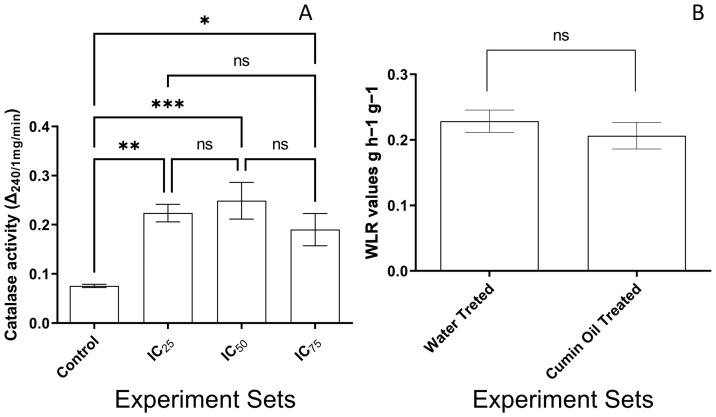
(**A**) Catalase activity in CSO treated *F. graminearum* PH-1 and (**B**) CSO response of *T. aestivum* L. cv. Ceyhan-99. ns: no significant changes, *: *p* < 0.05, **: *p* < 0.01, and ***: *p* < 0.001.

The effect of CSO on the metabolization of the oxygen was examined by determining CAT activity alterations in the *F. graminearum* PH-1 strain. The CSO treatment led to significant differences between the control and the experiment sets via one-way ANOVA analysis. The values related to CAT activity (Δ_240/1mg/min_), 0.07 ± 0.01, 0.22 ± 0.01, 0.24 ± 0.03 and 0.19 ± 0.03, were recorded in control, IC_25_ (*p* ˂ 0.01), IC_50_ (*p* ˂ 0.001), and IC_75_ (*p* ˂ 0.05) sets, respectively (Figure 4A).

### 3.6. WLR Analysis

No significant differences were detected between the WLR values of 14-day-old plantlets belonging to the control or the experiment sets (*p* > 0.05). Mean WLR values were recorded as 0.22 ± 0.01 and 0.20 ± 0.02 g h^−1^ g^−1^ for control and CSO treated sets (Figure 4B), respectively. No potential abiotic stress presence was detected due to CSO treatment in *T. aestivum* L. Ceyhan 99.

### 3.7. Statistical Analysis

**Figure 5:**
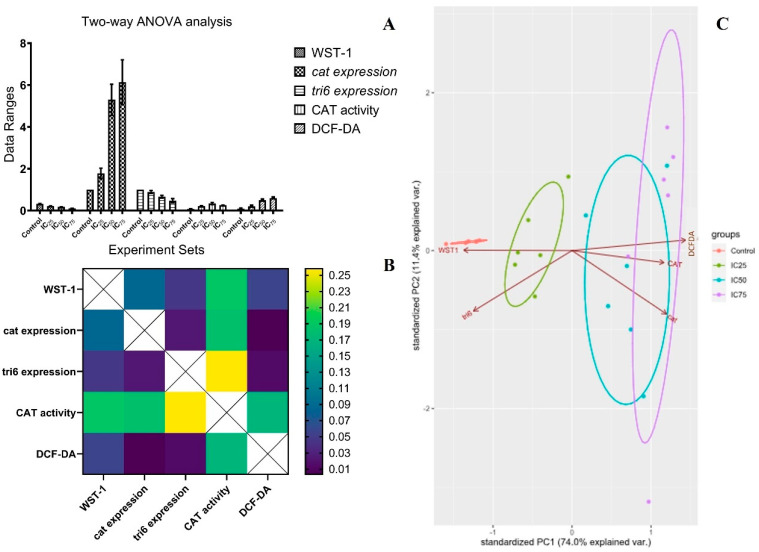
Two-way ANOVA analysis (**A**), *p* values dendrogram for correlation status (**B**), and PCA profiling (**C**) of different experimental procedures carried out in this study. Control, IC_25_, IC_50_, and IC_75_ sets were the strains grown on PDA amended with 0, 0.125, 0.25, and 0.375 µg µL^−1^ cumin seed oil.

Two-way ANOVA analyses were combined with Pearson’s correlation matrix in order to reveal a correlation between different experimental procedures. By this way, data obtained from the WST-1 assay, DCF-DA staining analysis, catalase activity, CAT expression, and tri6 expression analysis were co-evaluated (Figure 4). In Pearson’s correlation test, both negative and also positive correlations were found between different experimental procedures (Table 3). *p* Values were recorded between 0.004 and 0.258. The correlation efficiency values were detected between −0.986 and +0.955. It was clear that CAT activity and CAT expression presented a positive correlation. Similarly, positive correlation between cell viability and *tri6* expression was recorded in the two-dimensional analysis (PC1: 74%, PC2: 11.4%, proportional variance). In contrast, negative correlation was also found in gene expression analysis. In PCAs, the control, IC_25_, IC_50_, and IC_75_ sets were co-clustered in all different experimental procedures (Figure 5).

## 4. Discussion

Plant-derived essential oils mixtures or specific compounds could show antimicrobial effects due to their valuable components. Particularly, these compounds could be an important part of an antagonistic effect on fungal phytopathogens [39,40]. Up to now, many plant-derived essential oils, primarily as a methanol extracts, had been tested on *F. graminearum* or genetically closely related phytopathogenic fungal species in order to reveal their potential antifungal usage [28,39,41,42,43]. However, the previous studies mainly included revealing the composition of essential oil mixtures and determining the radial growth rate inhibition in fungi. The detailed and comprehensive data related to the mechanisms, which lie behind the potential antifungal activities of these plant-derived essential oils, are missing from the literature. Within this scope, the potential antifungal activity of cumin seed oil on physiological, genetics, epigenetics, and transcriptional levels of *F. graminearum* was evaluated.

Previous studies have shown that there are different ranges for inhibition values of radial growth rate in *Fusarium* spp. An approximately 50% growth inhibition due to aloe vera extracts treatment was recorded in *F. oxysporum* [42]. Similarly, 1 μg μL^−1^ *Haplophyllum tuberculatum* essential oil extracts treatment resulted in a 49% radial growth inhibition in *F. oxysporum*. Singh et al. [39] reported that *Foeniculum vulgare* oil mixtures up to 6 μL led to 100% growth inhibition in *F. graminearum*. Perczak et al. [28] tested the potential antifungal activities of different plant-derived essential oil mixtures on *F. graminearum* and *F. culmorum* and they showed that there was great variation among minimum inhibition concentrations of different plant-derived essential oils. In this study, a relatively very low level of CSO was needed to repress the radial growth of *F. graminearum*. The MIC value for CSO (0.5 mg mL^−1^) was also relatively low in comparison to specific plant-derived essential oil compounds, such as α-thujone and camphor [25,26,29]. Results obtained from this study yielded that the concentration to be used for in vitro tests of CSO is closely near to concentrations of specific fungicides [19,21,23,24]. In addition to radial growth rate analysis, the potential growth inhibitory effects of CSO were also confirmed by using cell proliferation tests related to the WST-1 assay in this study. The presence of plant-derived essential oils led to toxicity in *F. graminearum* as reported by Teker et al. [29].

In recent years, several reports showed that plant-derived essential oil compounds or specific fungicides led to a high level of genomic and epigenomic alterations in phytopathogenic fungi [23,25,29]. *HapI*I and *Msp*I enzymes were used in epigenomic analysis. *Hap*II digestion is active in type I (CCGG; second C is methylated) and type II (CCGG; first C is methylated) methylation, whereas *Msp*I enzyme is active for only type I and type III (CCGG; second C is methylated) methylation. Type IV (CCGG; first C is methylated) methylation includes no active digestion by *Hpa*II and *Msp*I [44,45]. By using RAPD and CRED-RA methods, the potential genomic stability changes and type I–IV methylation presence were aimed to be revealed in this study. However, only a limited and low level of genomic instability was detected by the GTS and RP analyses. Similarly, type II and type III methylation changes of up to 15% polymorphisms were detected in the IC_25_, IC_50_, and IC_75_ sets. A relatively stable methylation status was detected in experimental sets in comparison to previous studies, including the usage of specific plant-derived essential oil compounds [25,29]. This low level of genomic and epigenomic changes due to CSO treatment (in comparison to specific plant-derived essential oil compounds or common fungicides) could arise from the complex or comprehensive content of the CSO, and some of the compounds present in the mixture could be effective in genomic and epigenomic stabilization. Further studies could include the effects of major and minor compounds of CSO mixtures on phytopathogenic fungi in terms of genomic or epigenomic alterations.

The changes in the expression of tri6 gene encoding the transcription factor of zinc finger motif related to trichothecene biosynthesis was also investigated in this study. Increased concentrations of CSO yielded a decreased expression of the *tri6* gene. Down-regulation up to 54% in tri6 expression was recorded due to CSO treatment. This decrease in trichothecene biosynthesis-related gene expression was accordant with previous studies, including essential oil compound and fungicide treatment against *Fusarium* spp. [23,24,25,29]. Moreover, negative correlations between tri6 expression and DCF staining and CAT expression were recorded. The findings in the current study provide preliminary data that CSO could be a potential repressor of trichothecene biosynthesis. However, in planta studies including CSO treatment could be useful for providing more detailed and comprehensive data in the near future.

The presence and adverse effects of oxidative stress in phytopathogenic fungi is one of the most important topics in plant pathology. Recent investigations showed that the presence and the level of oxidative stress in fungi (exposed to the stress factor[s]) affect important physiological processes such as mycotoxin biosynthesis and apoptosis destination in fungi [29,46,47,48,49]. In this study, potential oxidative stress presence in *F. gramineraum* in response to CSO treatment was investigated by qPCR, DCF-DA staining, and CAT activity assays. In comparison to previous studies, a moderate to high level of oxidative stress was detected via CSO treatment [2,29,48,49,50]. Each method independently showed that CSO treatment led to oxidative stress in the experiment sets. Moreover, CAT expression and the DCF-DA staining analysis were found to be positively correlated by posterior statistical analysis. The findings obtained from this study show that CSO strongly shows antifungal activities and represses radial growth on *F. graminearum* by oxidative stress in vitro for a limited part of the lifetime of fungi. Detailed antifungal activity of CSO or other plant-derived compounds on *F. graminearum* could be evaluated in terms of mycotoxin production and pigmentation processes.

This study, including data related to the hemi-biotrophic worldwide phytopathogen *F. graminearum,* provides preliminary and informative data for conducting similar studies in other fungal genera. The output of the study is of great importance in terms of revealing the potential of a new and natural antifungal compound that can be used in the fight against *Fusarium* spp. and related fungal phytopathogens’ diseases.

## 5. Conclusions

Synthetic antifungal products, particularly common fungicides, have been used to manage and combat fungal diseases worldwide. However, they have detrimental effects on the environment such as the presence of fungicide resistant isolates and the accumulation of toxic waste. To avoid these undesirable effects, natural antifungal compounds are needed. This is the first report including physical, genetics, epigenetics, and transcriptional levels of investigations that show CSO could be considered as a potential antifungal agent against phytopathogenic fungi. Findings obtained from this study show that cumin seed oil overcomes, in particular, *F. graminearum* by oxidative stress formation in vitro. Further studies need in field investigations and mammalian culture tests to detect potential adverse effects of CSO on useful microorganisms and/or animals.

## Figures and Tables

**Table 1:** RAPD primers and total amplicon numbers obtained from analysis.

Primer	Primer Sequence (5′-3′)	Total Band Number	Polymorphic Band Number	Polymorphic Band %	RP Values
OPA04	AATCGGGCTG	6	5	0.83	3.3
OPA05	AGGGGTCTTG	4	3	0.75	1.98
OPA09	GGGTAACGCC	7	6	0.85	3.64
OPA13	CAGCACCCAC	6	4	0.66	2.64
OPB06	TGCTCTGCCC	4	3	0.75	1.98
OPB07	GGTGACGCAG	5	4	0.8	2.64
OPB09	TGGGGGACTC	8	7	0.875	4.62
OPB10	CTGCTGGGAC	6	4	0.66	2.48
OPB13	TTCCCCCGCT	4	4	1	2.64
OPB19	CCGCATCTAC	4	4	1	2.64
OPC04	CCGCATCTAC	4	4	1	2.64
OPC05	GATGACCGCC	6	4	0.66	2.64
OPC07	GTCCCGACGA	5	4	0.8	2.64
OPG13	CTCTCCGCCA	4	3	0.75	1.98
OPG16	AGCGTCCTCC	4	2	0.5	1.32
OPM01	GTTGGTGGCT	7	6	0.85	3.84
OPM02	ACAACGCCTC	11	10	0.90	5.74
OPM03	GGGGGATGAG	8	7	0.875	4.54
OPM04	GGCGGTTGTC	10	9	0.9	5.38
OPM05	GGGAACGTGT	9	8	0.88	4.68
OPM07	CCGTGACTCA	7	7	1	3.52
OPM09	GTCTTGCGGA	6	5	0.83	3.04
OPM10	TCTGGCGCAC	6	5	0.83	3.04

**Table 2:** CRED-RA results obtained in *F. graminearum* PH-1 strain.

Primer	Control		Total Number of Experimental Band		Total Number of Experimental Band		Total Number of Experimental Band		Total Number of Experimental Polymorphic Band		Total Number of Experimental Polymorphic Band		Total Number of Experimental Polymorphic Band		Polymorphism (%)		Polymorphism (%)		Polymorphism (%)	
			IC_25_		IC_50_		IC_75_		IC_25_		IC_50_		IC_75_		IC_25_		IC_50_		IC_75_	
	*Hap*II	*Msp*I	*Hap*II	*Msp*I	*Hap*II	*Msp*I	*Hap*II	*Msp*I	*Hap*II	*Msp*I	*Hap*II	*Msp*I	*Hap*II	*Msp*I	*Hap*II	*Msp*I	*Hap*II	*Msp*I	*Hap*II	*Msp*I
OPA04	4.0	4.0	4.0	4.0	4.0	4.0	4.0	4.0	0.0	0.0	0.0	0.0	0.0	0.0	0.0	0.0	0	0	0	0.0
OPA05	2.0	2.0	2.0	2.0	2.0	2.0	2.0	2.0	0.0	0.0	0.0	0.0	0.0	0.0	0.0	0.0	0	0	0	0.0
OPA09	2.0	3.0	2.0	2.0	2.0	4.0	4.0	3.0	0.0	1.0	0.0	1.0	2.0	0.0	0.0	50.0	0	25	50	0.0
OPA13	3.0	3.0	3.0	3.0	3.0	3.0	3.0	3.0	0.0	0.0	0.0	0.0	0.0	0.0	0.0	0.0	0	0	0	0.0
OPB06	2.0	2.0	2.0	2.0	2.0	2.0	2.0	2.0	0.0	0.0	0.0	0.0	0.0	0.0	0.0	0.0	0	0	0	0.0
OPB07	1.0	1.0	1.0	1.0	1.0	1.0	1.0	1.0	0.0	0.0	0.0	0.0	0.0	0.0	0.0	0.0	0	0	0	0.0
OPB09	7.0	2.0	7.0	2.0	7.0	2.0	7.0	2.0	0.0	0.0	0.0	0.0	0.0	0.0	0.0	0.0	0	0	0	0.0
OPB10	6.0	5.0	6.0	4.0	6.0	5.0	6.0	5.0	0.0	1.0	0.0	0.0	0.0	0.0	0.0	25.0	0	0	0	0.0
OPB13	1.0	0.0	1.0	0.0	1.0	0.0	1.0	0.0	0.0	0.0	0.0	0.0	0.0	0.0	0.0	0.0	0	0	0	0.0
OPB19	1.0	0.0	1.0	0.0	1.0	0.0	1.0	0.0	0.0	0.0	0.0	0.0	0.0	0.0	0.0	0.0	0	0	0	0.0
OPC04	1.0	0.0	1.0	0.0	1.0	0.0	1.0	0.0	0.0	0.0	0.0	0.0	0.0	0.0	0.0	0.0	0	0	0	0.0
OPC05	4.0	4.0	4.0	4.0	4.0	4.0	4.0	4.0	0.0	0.0	0.0	0.0	0.0	0.0	0.0	0.0	0	0	0	0.0
OPC07	3.0	2.0	3.0	2.0	3.0	2.0	3.0	2.0	0.0	0.0	0.0	0.0	0.0	0.0	0.0	0.0	0	0	0	0.0
OPG13	1	1	1	1	1	1	1	1	0	0	0	0	0	0	0.0	0.0	0	0	0	0.0
OPG16	3	3	3	3	3	3	3	3	0	0	0	0	0	0	0.0	0.0	0	0	0	0.0
OPM01	7.0	7.0	7.0	6.0	6.0	4.0	6.0	4.0	0.0	0.0	1.0	3.0	1.0	3.0	0.0	0.0	16.66	75	16.66	75.0
OPM02	6.0	6.0	6.0	6.0	4.0	7.0	6.0	3.0	0.0	0.0	2.0	1.0	0.0	3.0	0.0	0.0	50	14.28	0	100.0
OPM03	3.0	4.0	4.0	2.0	5.0	2.0	4.0	4.0	1.0	2.0	2.0	2.0	1.0	0.0	25.0	100.0	40	100	25	0.0
OPM04	7.0	4.0	7.0	3.0	6.0	2.0	6.0	3.0	0.0	1.0	1.0	2.0	1.0	1.0	0.0	33.3	16.66	100	16.66	33.3
OPM05	4.0	3.0	5.0	4.0	7.0	3.0	6.0	5.0	1.0	1.0	3.0	0.0	2.0	2.0	20.0	0.0	42.85	0	33.33	40.0
OPM07	1.0	1.0	2.0	1.0	4.0	2.0	4.0	2.0	1.0	0.0	3.0	1.0	3.0	1.0	50.0	0.0	75	50	75	50.0
OPM09	2	1	1	1	2	1	2	1	1	0	0	0	0	0	100.0	0.0	0	0	0	0.0
OPM10	4	3	3	3	4	3	3	3	1	0	0	0	1	0	33.3	0.0	0	0	33.33	0.0
	3.3	2.7	3.3	2.4	3.4	2.5	3.5	2.5	0.2	0.3	0.5	0.4	0.5	0.4	9.9	9.1	10.5	15.8	10.9	13.0

**Table 3:** Pearson correlation matrix between experimental tests. Numeric data in columns are of Pearson r values. ^+^: positive correlation and ^−^: negative correlation.

	WST-1	*cat* Expression	*tri6* Expression	CAT Activity	DCF-DA
WST-1	1.00	−0.85	0.71	−0.96	−0.85
*CAT* expression	−0.85	1.00	−0.24	0.96	0.99
*tri6* expression	0.71 ^+^	−0.24 ^−^	1.00	−0.49	−0.24
CAT activity	−0.96	0.96	−0.49	1.00	0.96
DCF-DA	−0.85	0.99 ^+^	−0.24 ^−^	0.96	1.00

## Data Availability

The original contributions presented in the study are included in the article, further inquiries can be directed to the corresponding author.
